# Breast cancer cell invasion mediated by Gα12 signaling involves expression of interleukins-6 and −8, and matrix metalloproteinase-2

**DOI:** 10.1186/1750-2187-9-6

**Published:** 2014-06-17

**Authors:** Crystal Y Chia, Udhaya Kumari, Patrick J Casey

**Affiliations:** 1 Program in Cancer and Stem Cell Biology, Duke-NUS Graduate Medical School, 8 College Road, Singapore 169857, Singapore

**Keywords:** G protein, GNA12, GNA13, Metastasis, Cytokine, NF-κB, Transcription

## Abstract

**Background:**

Recent studies on the involvement of the G12 family of heterotrimeric G proteins (Gα12 and Gα13, the products of the GNA12 and GNA13 genes, respectively) in oncogenic pathways have uncovered a link between G12 signaling and cancer progression. However, despite a well characterized role of Rho GTPases, the potential role of secreted factors in the capacity of G12 signaling to promote invasion of cancer cells is just beginning to be addressed.

**Methods:**

MDA-MB-231 and MCF10A breast cancer cell lines were employed as a model system to explore the involvement of secreted factors in G12-stimulated cell invasion. Factors secreted by cells expressing dominant-active Gα12 were identified by protein array, and their involvement in breast cancer cell invasion was assessed through both RNAi-mediated knockdown and antibody neutralization approaches. Bioinformatics analysis of the promoter elements of the identified factors suggested NF-κB elements played a role in their enhanced expression, which was tested by chromatin immunoprecipitation.

**Results:**

We found that signaling through the Gα12 in MDA-MB-231 and MCF10A breast cancer cell lines enhances expression of interleukins (IL)-6 and −8, and matrix metalloproteinase (MMP)-2, and that these secreted factors play a role in G12-stimulated cell invasion. Furthermore, the enhanced expression of these secreted factors was found to be facilitated by the activation of their corresponding promoters, where NF-κB seems to be one of the major regulators. Inhibition of IL-6 and IL-8, or MMP-2 activity significantly decreased Gα12-mediated cell invasion.

**Conclusions:**

These studies confirm and extend findings that secreted factors contribute to the oncogenic potential of G12 signaling, and suggest potential therapeutic targets to control this process.

## Background

Heterotrimeric guanine nucleotide-binding proteins (G proteins) mediate extracellular signals from transmembrane G protein-coupled receptors (GPCRs) to activate intracellular effector molecules, triggering signaling pathways that lead to a variety of cellular responses [1,2]. G proteins consist of two functional units, a guanine nucleotide-binding α subunit and a βγ subunit dimer, and are classified according to their α-subunit into four subfamilies: Gs, Gi, Gq, and G12 [2].

Cancer invasion and metastasis are two major challenges for cancer treatment, and are usually the cause of an overall decrease in the long-term survival rates of cancer patients. Studies have suggested a role for members of the G12 family, comprised of the α-subunits Gα12 and Gα13, in mediation of signaling pathways related to cell growth and tumorigenesis [3-5]. Our previous work showed that high levels of Gα12 and Gα13 protein expression had a direct correlation with the severity of grade of breast and prostate tumors, and activation of these proteins promoted cell migration and invasion [6,7]. Further examination of the role of G12 signaling in cancer cell invasion and tumor progression has highlighted the potential impact of this process on a number of tumor types [8,9]. These findings have provided compelling evidence that G12 signaling is an important regulator of cancer cell invasion and metastasis.

The finding that activation of G12 proteins promotes cancer cell migration and invasion has highlighted the need to understand the biologic processes underlying this phenomenon. Numerous studies have identified the GTPase Rho as a major mediator of G12 signaling via its ability to effect changes in cytoskeletal dynamics required for cell migration, invasion and engagement of the cellular transcriptional machinery [4,10]. Both the transforming ability and the impact on cell invasion and metastasis of G12 proteins appear to be dependent, at least in part, on their ability to engage Rho GTPases. However, oncogenesis and cell migration and invasion are complex processes, and in some cell types G12 proteins promote these activities independent of, or via processes acting in conjunction with Rho activation [11,12]. Additionally, evidence implicating G12 proteins in activation of NF-κB and secretion of IL-8 [13], and up-regulation of metalloproteinase MMP-2 [14,15], suggests that other downstream effectors of G12 proteins may also be important in their biological activities.

We report here that, in breast cancer cell lines, the ability of activated Gα12 to promote cell invasion is highly dependent on its capacity to promote expression and secretion of interleukins and MMP-2. The increased expression of MMP-2 through Gα12 activation has been shown in an earlier study [14], and our results recapitulate their findings. In the current study, we further show that this activity is highly dependent on activation of the corresponding promoter elements of these genes, with NF-κB playing a major role. Inhibition of interleukin or MMP-2 activity significantly decreased Gα12-mediated cell invasion. These results identify novel factors that are important for the ability of G12 signaling to promote biological processes involved in tumor progression.

## Results

### Secreted factors are involved in the ability of dominant active Gα12 to induce invasion of breast cancer cells

**Figure 1:**
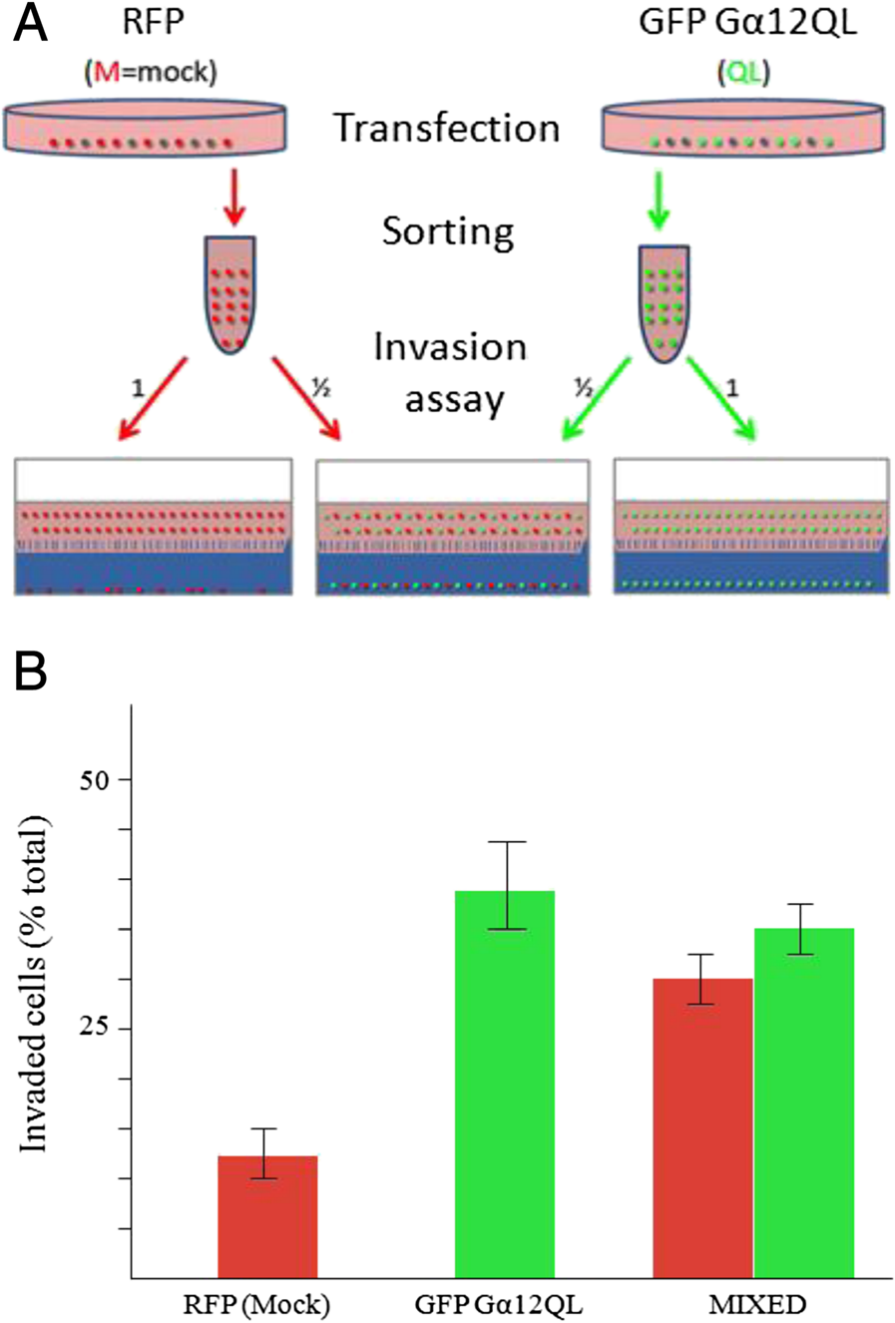
**Dominant active Gα12 increases invasiveness of MDA-MB-231 breast cancer cells via paracrine signaling. (A)** Experimental scheme of the *in vitro* invasion assay to test hypothesis that MDA-MB-231 cells transfected with Gα12QL drive the invasion of neighboring untransfected cells. Following transfection with the indicated vectors, cells were sorted and enriched fractions of RFP/mock (M) or GFP/Gα12QL (QL) containing cells (each at ~98% purity), or a 1:1 mixture of these cells, were subjected to the invasion assay followed by FACS analyses. **(B)** Results from FACS analysis showing invasion of the population of RFP/mock, GFP/Gα12QL and a mixed population of both RFP/mock and GFP/Gα12QL cells. Invaded cells were counted and plotted as a percentage of total cells subjected to the analysis. Values are plotted as the mean ± S.E. The results are from a single experiment that is representative of three independent experiments.

**Figure 2:**
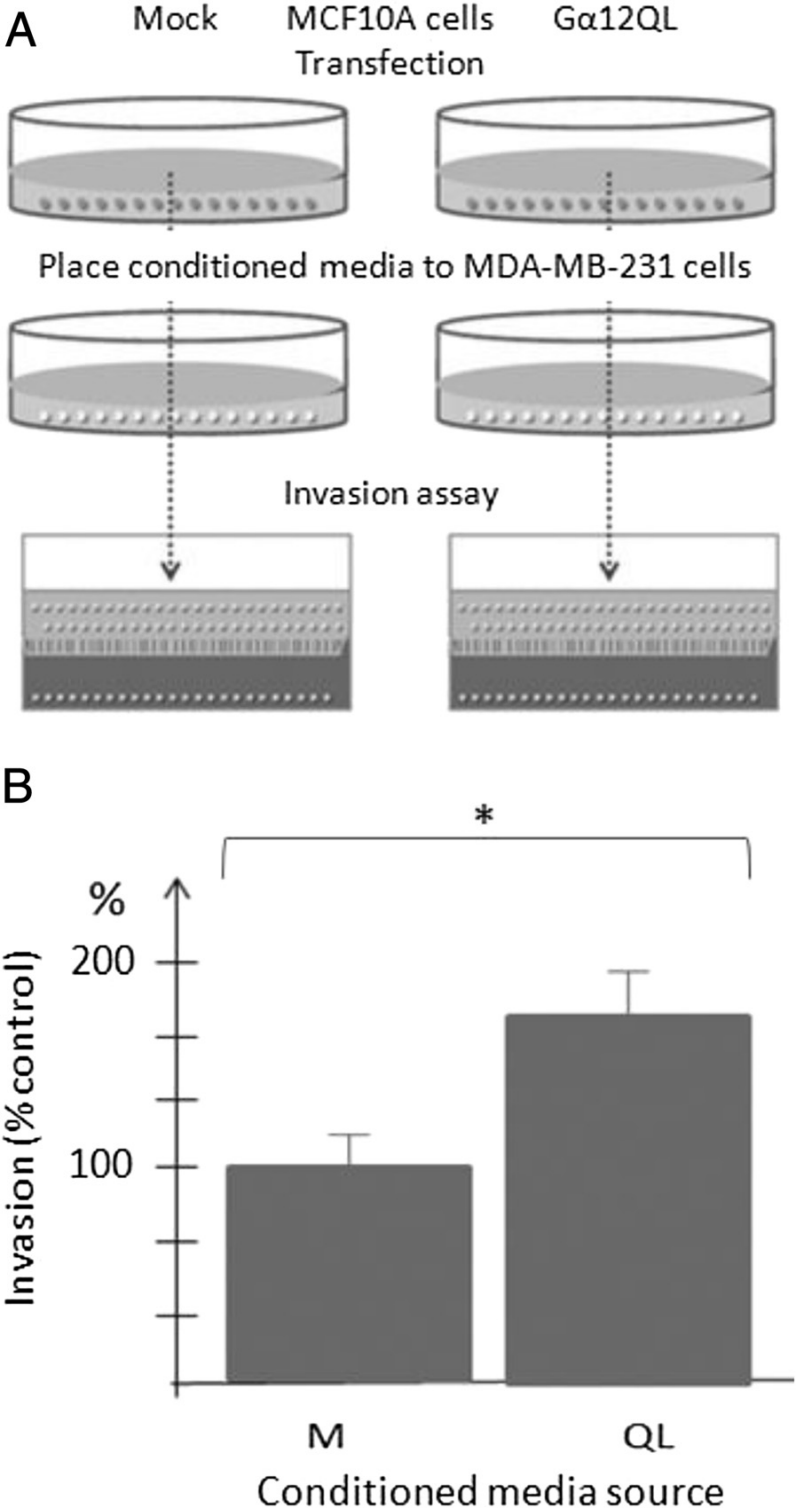
**Factors secreted from MCF10A cells expressing dominant active Gα12 stimulate the invasion of MDA-MB-231 cells. (A)** Experimental scheme illustrating experimental conditions. MCF10A cells were transfected as described under “Methods”. Following a 48 h incubation period, the conditioned media was collected and placed on MDA-MB-231 cells in 6 well plates. After 12 h, the MDA-MB-231 cells were harvested and subjected to an invasion assay. The results are shown in **(B)**. Data are presented as a mean of triplicate determinations from a single experiment that is representative of two independent experiments. Bars represent the mean ± S.E. *p < 0.05.

A number of studies have indicated that activation of Gα12 signaling pathways leads to an increase in cell invasion [6,7,9]. In the course of performing such studies with MDA-MB-231 and MCF10A breast cancer cells, we found that the enhancement in the number of invading cells upon introduction of dominant-active Gα12 (Gα12QL) was much higher than the transfection efficiency of Gα12 construct (data not shown). This observation suggested that Gα12QL-expressing cells were likely influencing the behavior of their non-transfected neighbors i.e. that activated G12 was triggering the production and/or release of paracrine factors that were promoting the invasion of non-transfected neighboring cells. To test this hypothesis, MDA-MB-231 breast cancer cells were transfected with either a RFP empty vector (mock) or with a bicistronic vector producing both Gα12QL and GFP. The cells were then sorted according to the expression of RFP or GFP, resulting in enriched fractions that contained >95% red or green cells. Invasion assays were then performed with individual red or green cell populations or with a 1:1 mixture of red and green cells, where the green cells were also expressing Gα12QL (experimental scheme shown in Figure 1A). As expected the GFP/Gα12QL cells showed a roughly 3-fold enhancement of invasion compared to the RFP/mock cells (Figure 1B). However, when the cells expressing either RFP or GFP/Gα12QL were mixed, both populations showed enhanced invasion, with the RFP-mock cells invading almost as well as the GFP/Gα12QL cells (Figure 1B), even though the former were not expressing Gα12QL.To further test the hypothesis that secreted factors were involved in promoting Gα12-stimulated cell invasion, we prepared conditioned media from MCF10A cells transfected with either control vector or that expressing Gα12QL. An invasion assay was then performed with non-transfected MDA-MB-231 cells exposed to the two types of conditioned media from the MCF10A cells (see Figure 2A for experimental scheme). The results of this assay, shown in Figure 2B, revealed a clear increase in the invasiveness of the MDA-MB-231 cells exposed to conditioned media from the cells expressing Gα12QL as compared to media from the cells expressing the vector control. Together, these findings provided compelling evidence that Gα12QL expressing cells were secreting factors that promote cell invasiveness.

### Activated Gα12 increases secretion of select cytokines and a matrix metalloprotease

**Figure 3:**
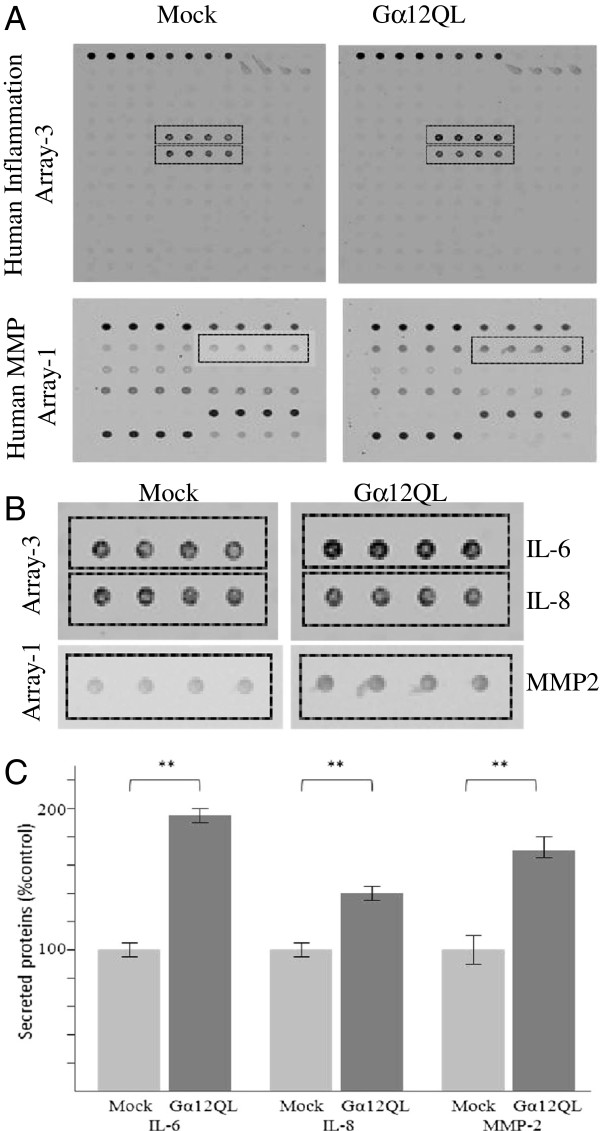
**Expression of dominant active Gα12 in MDA-MB-231 cells induces secretion of cytokines IL-6 andIL-8, and MMP-2. (A)** Protein array analysis of factors present in conditioned media. MDA-MB-231 cells were transfected either with control vector (Mock) or Gα12QL as indicated. Following a 72 h incubation period, media was harvested and subject to antibody-based arrays; see Methods for details. **(B)** Enlarged areas of the indicated regions of the arrays shown in **(A)**. Quantification of data from three arrays is shown in **(C)**. Bars represent the mean ± S.E. of quadruplicate determinations; IL-6 (p = 0.007), IL-8 (p = 0.003), MMP-2 (p = 0.007).

To identify factors whose secretion was enhanced by expression of Gα12, we utilized protein array assays to screen a panel of potential candidates, including 40 cytokines, MMPs and MMP inhibitors (Figure 3A, B). Conditioned media from MDA-MB-231 and MCF10A cells expressing either vector control or Gα12QL was harvested, and the levels of the various factors represented on the arrays determined via ELISA (Additional file 1: Figures S1 – S3). This analysis revealed significantly increased levels of IL-6, IL-8 and MMP-2 in conditioned media from the cells expressing Gα12QL; the primary data for MDA-MB-231 and MCF10A cells and its quantitation are shown in Figure 3 and Additional file 1: Figure S4 respectively.

**Figure 4:**
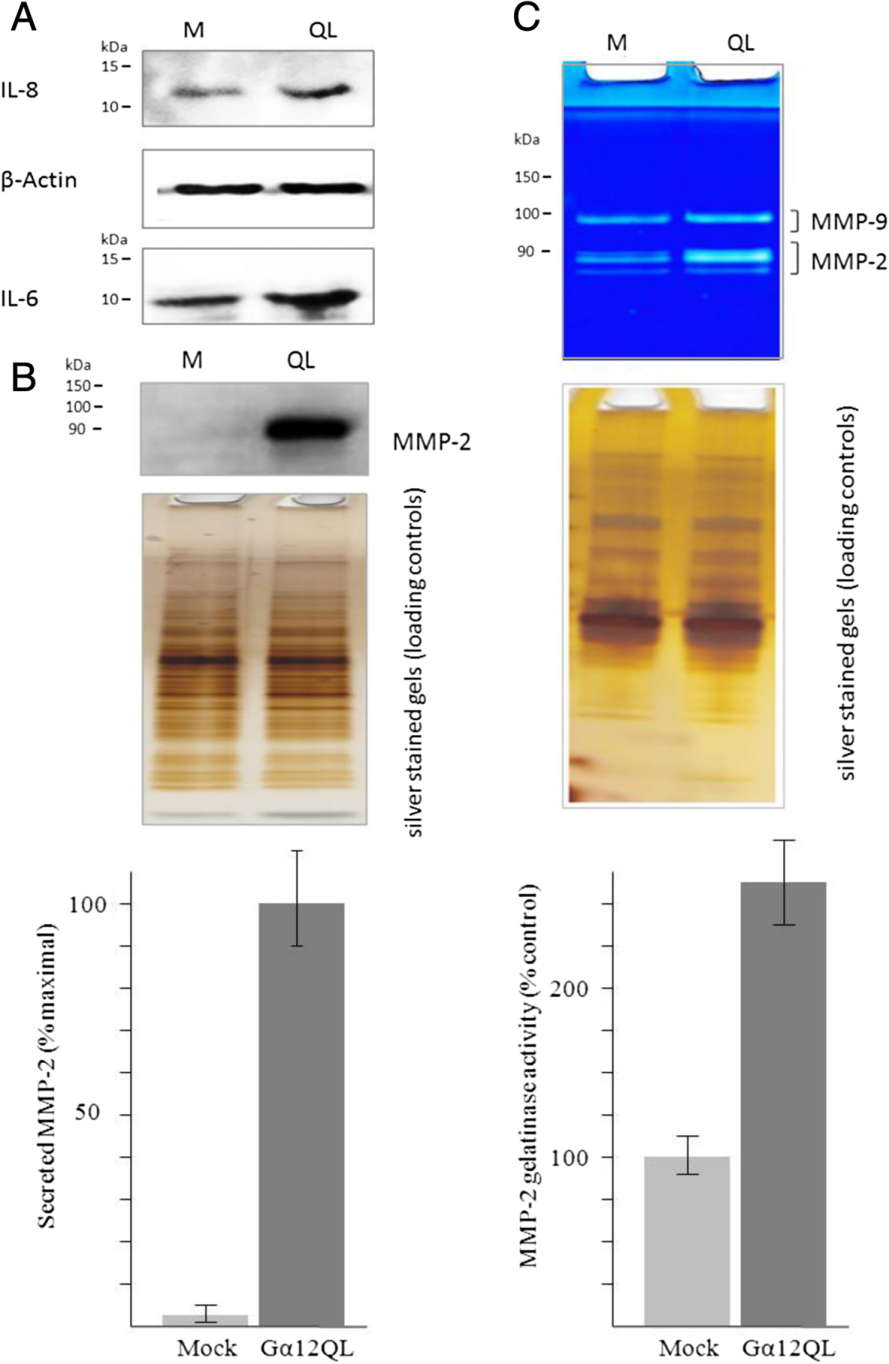
**Validation of increased secretion of IL-6, IL-8 and MMP-2 expression of MDA-MB-231 cells expressing dominant activate Gα12. (A)** Expression of IL-6 and IL-8 in total cell lysates of MDA-MB-231 cells transfected with either mock (M) vector or Gα12QL (QL) vector. MDA-MB-231 cells were transfected with the indicated construct and cell lysates prepared 72 h later and subject to immunoblot analysis as described under Methods. **(B)** MMP-2 levels in conditioned media of MDA-MB-231 cells transfected with either mock (M) vector or Gα12QL (QL) vector. MDA-MB-231 cells were transfected with the indicated construct and media harvested 72 h later and subject to immunoblot analysis as described under Methods. **(C)** Zymography analysis of gelatinase activity of MMP-2 in conditioned media of MDA-MB-231 cells transfected with either mock (M) vector or Gα12QL (QL) vector. Experimental conditions are as in **(B)**. In the center panel, aliquots of conditioned media from were analyzed by silver-as the loading controls. Data in the lower panel are the mean ± S.E. of quadruplicate determinations from a single experiment that is representative of two independent experiments.

To validate the protein array results, we determined the levels of the IL-6 and IL-8 by immunoblot analysis of total cell lysates. In MDA-MB-231 cells transfected with vector Gα12QL, an increase in both IL-6 and IL-8 was observed in the cells expressing Gα12QL (Figure 4A). In MCF10A cells, an increase in IL-8 was observed in the cells expressing Gα12QL (Additional file 1: Figure S5A). As for IL-6 levels in MCF10A cells, we were unable to detect a signa. We also attempted to detect the secretion of both interleukins in the culture media of both cell lines via immunoblot analysis, but their levels were apparently below the detection limit of the antibodies we had available.

To confirm the impact of Gα12QL expression on MMP-2 secretion, immunoblot analysis of the culture media of MDA-MB-231 and MCF10A cells expressing vector or Gα12QL was performed. In concordance with a previous report [14], expression of the activated Gα12 protein led to a substantial increase in MMP-2 in both MDA-MB-231 (Figure 4B) and MCF10A culture media (Additional file 1: Figure S5B). We also tested the conditioned media of both the MDA-MB-231 and MCF10A cells for the expression of MMP-1, MMP-3, MMP-7, MMP-8 and MMP-9, but found no significant difference in response to Gα12QL expression (data not shown). To confirm that there was an actual increase in MMP-2 activity in the media of cells expressing Gα12QL, zymogram analyses were performed. Quantitation of this analysis showed increased MMP-2 activity in conditioned media of MDA-MB-231 (Figure 4C) and MCF10A cells (Additional file 1: Figure S5C). *In-situ* zymography was then performed to assay the activity of secreted MMP-2 from living MDA-MB-231 cells (Additional file 1: Figure S6). Indeed, Gα12QL-expressing cells displayed a higher gelatinase activity. Co-transfection of MDA-MB-231 cells with siRNA targeting MMP-2 significantly reduced gelatinase activity in the media of Gα12QL-expressing cells, confirming that this gelatinase activity was due to the increased secretion of MMP-2.

### Impairment of IL-6, IL-8 and MMP-2 function decreases Gα12QL-stimulated breast cancer cell invasion

**Figure 5:**
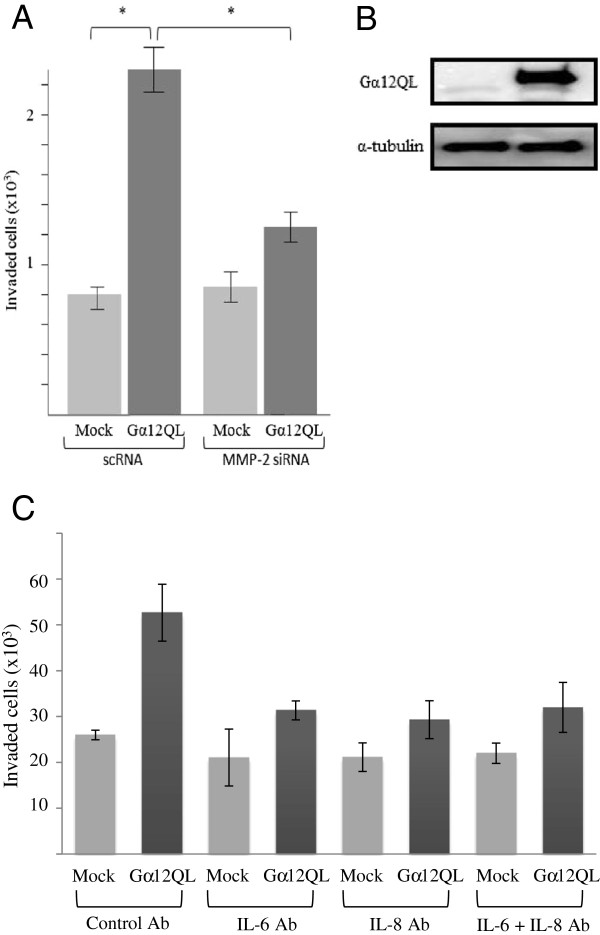
**Interleukins and MMP-2 are involved in Gα12-mediated invasion of MDA-MB-231 cells.** MDA-MB-231 cells were transfected with either control vector (Mock) or Gα12QL vectors as indicated. In addition, for **(A)** the cells were co-transfected with either scrambled (scRNA) or MMP-2 siRNA; the plasmid vectors also expressed GFP. The green cells were then sorted, and the enriched fractions were subjected to invasion assays. Depletion of MMP-2 with specific siRNA decreases Gα12-stimulated invasion of MDA-MB-231 cells. Data is pooled from three independent experiments, each involving duplicate determinations. Bars represent the mean ± S.E.; *(p < 0.05). **(B)** Representative western blot showing the over-expression of Gα12QL after doxycycline induction in MDA-MB-231 stable cell line. **(C)** Addition of IL-6 and IL-8 specific antibodies to the media of MDA-MB-231 cells stably expressing Gα12QL impairs Gα12-stimulated cell invasion. Data is pooled from three independent experiments, each involving triplicate determinations. Bars represent the mean ± S.E.

To assess the role of MMP-2 in Gα12QL-stimulated breast cancer cell invasion, we employed both genetic and pharmacologic suppression of the enzyme. Knockdown of MMP-2 in MDA-MB-231 cells (Figure 5A) and MCF10A cells (Additional file 1: Figure S7), or treatment of the cells with MMP inhibitor GM6001 (data not shown), dramatically impacted Gα12QL-stimulated cell invasion These results clearly demonstrated that MMP-2 plays a major role in breast cancer cell invasion stimulated by G12 activation, confirming previous observations with MCF10A cells [14].To assess the importance of the enhanced secretion of IL-6 and IL-8 in cancer cell invasion stimulated by Gα12QL, we neutralized these secreted cytokines using monoclonal antibodies. For these experiment we used MDA-MB-231 cells stably expressing Gα12QL. Neutralization of secreted IL-6 and IL-8 in the media of these cells significantly decreased their capacity to invade (Figure 5B). These data provide compelling evidence for a role for these cytokines in Gα12-stimulated cell invasion.

### Gα12QL stimulates IL-6, IL-8 and MMP-2 promoter activities via NF-κB binding

**Figure 6:**
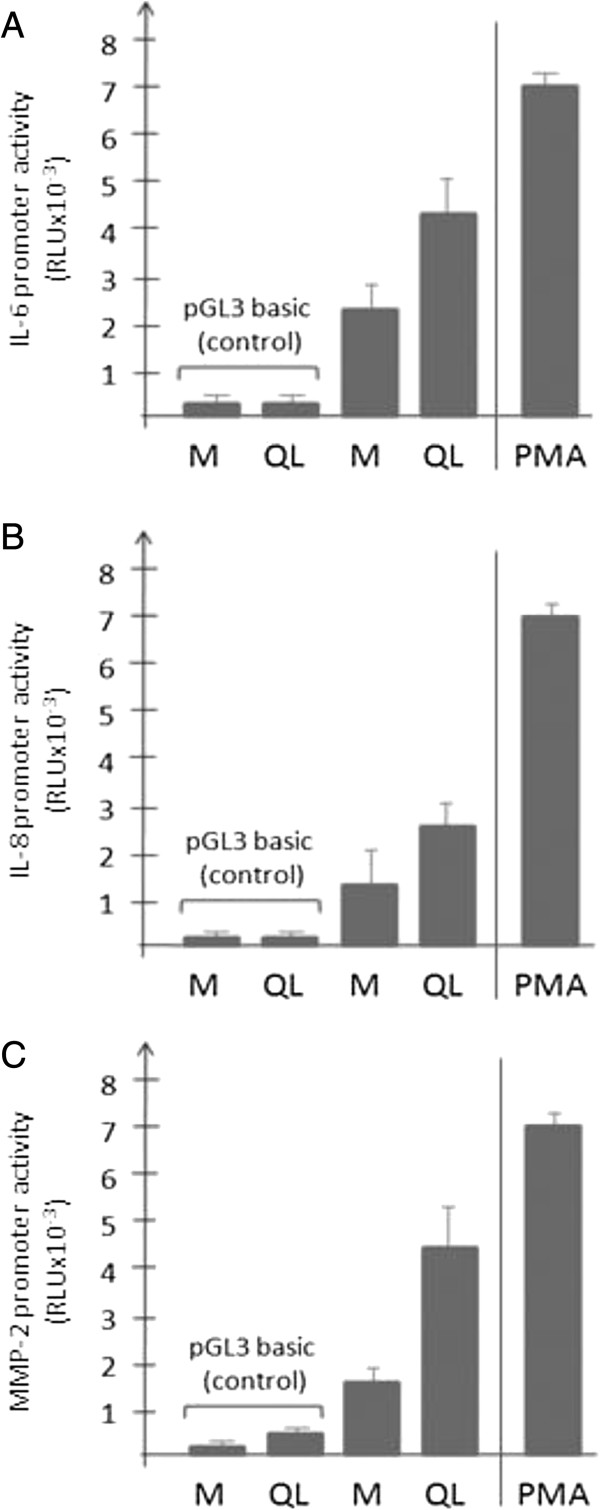
**Ectopic expression of dominant active Gα12 in MDA-MB-231 cells enhances promoter activities of IL-6, IL-8 and MMP-2.** Luciferase-based assays were conducted to analyze the impact of Gα12QL expression on the activities of an IL-6-Luc promoter **(A)**, IL-8-Luc promoter construct **(B)**, and a MMP-2-Luc promoter construct. **(C)** Mock (M) or Gα12QL expression vectors (200 ng of DNA per well of a 24-well plate) were co-transfected into the MDA-MB-231 cells along with the IL-6-Luc, IL-8-Luc or MMP-2-Luc vectors respectively, as well as pRL-TK (renilla control) as described in Methods. Induction of promoter activity by dominant active Gα12 expression or 10 ng/ml PMA (positive control) was assessed for the respective constructs, corrected for transfection efficiency, and calculated as percentage of induction compared with unstimulated baseline activity. Data shown were pooled from three independent experiments, each with triplicate determinations. Bars represent the mean ± S.E.

**Figure 7:**
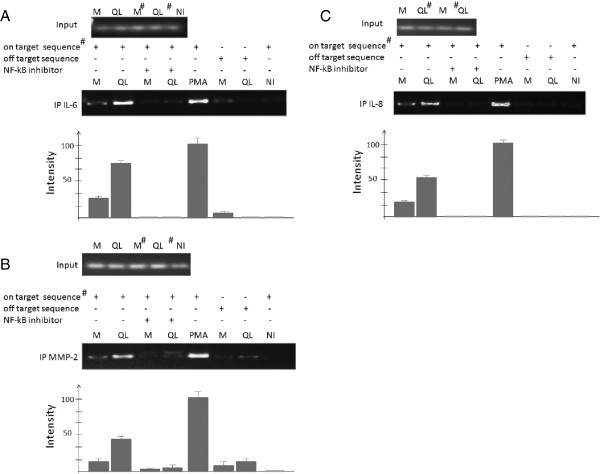
**ChIP assays reveals Gα12QL-dependent increase of NF- *κ *B occupancy of IL-6, IL-8, and MMP-2 promoters.** ChIP assays were performed on chromatin isolated from MDA-MB-231 cells transfected with mock (M) or Gα12QL vector expression vectors. Samples were pre-cleared by a precipitation protocol using control IgG, and then subjected to immunoprecipitation with anti-NF-κB. Semi-quantitative PCR was performed with primers targeted to a consensus NF-κB binding site upstream of the 5′-end of the corresponding genes. The sequences of neighboring regions were used as the “off target” sequence. The quantitative data reflect occupancy and represent mean ± SE of three independent experiments.

To begin to explore the molecular mechanisms underlying the ability of activated Gα12 to enhance expression and secretion of paracrine factors cancer cells, we investigated the promoter activities of the genes for IL-6, IL-8 and MMP-2. Expression of Gα12QL in MDA-MB-231 cells led to an increase in activity of all three promoter elements (Figure 6). Moreover, inactivation of Rho protein function by co-expression of C3 toxin essentially eliminated the responses to Gα12QL (data not shown). Since all three promoter elements contained consensus NF-κB binding sites (see Additional file 1: Figure S8), and stimulation of G12 proteins and RhoA has been reported to promote the activation of NF-*κ*B-regulated transcription [13,16], we focused our attention on a potential role for NF-*κ*B in this process. Hence, chromatin immunoprecipitation (ChIP) experiments employing an antibody targeting NF-*κ*B were performed using all three promoter elements. Comparison of the resulting DNA fragments from these promoter elements that immunoprecipitated with NF-κB clearly indicated increased occupancy of the promoter elements for IL-6, IL-8, and MMP-2 in the cells expressing Gα12QL compared with vector controls (Figure 7A,B, and C). This increase was not related to loading differences or variations in NF-κB expression in the cell samples, as demonstrated by the immunoblot analysis of cell lysates (not shown). Moreover, treatment of the cells with a NF-κB-specific inhibitor completely abolished the difference in signals between the Gα12QL-expressing and control cells (Figure 7A,B, and C), suggesting specificity of NF-κB binding to the corresponding DNA regions. Thus, these data indicate that activated Gα12 stimulates promoter activities of IL-6, IL-8 and MMP-2, and that NF-κB binding plays a major role in this stimulation.

## Discussion

The seminal study of Chan et al. provided the first evidence that elevated activity of Gα12 could contribute to tumorigenesis [17]. Subsequent studies uncovered Rho-specific guanine nucleotide exchange factors as critical mediators of G12 signaling, and provided a direct link to control of Rho protein activity [18-21]. In addition, G12 proteins likely engage several other regulatory pathways, as many proteins have been shown to bind Gα12 and Gα13 in an activation-sensitive fashion and participate in biologies that are dependent on G12/13 signaling [3,22-24]. Given recent studies identifying the G12 family proteins as important regulators of cancer cell invasion and tumor progression [6-9], determining the roles of different Gα12-mediated signaling processes in the responses to activation of this key G protein are critical to understanding its signaling network.

There has been several studies in recent years implicating G proteins in secretion and/or activity of cytokines and matrix metalloproteinases. These include findings of a direct association between Gαi and IL-8 receptors [25], and a role for Gαq, Gα12 and Gα13 in induction IL-6 via thromboxane A2 receptors in astrocytoma cells [26]. Secretion of IL-6 and IL-8 in dendritic cells was shown to be mediated by Gi proteins [27], while in osteoblasts, expression of constitutively-active Gα12 and Gα13 activated phospholipase D [28] and PLD-dependent signaling also contributes to IL-6 promoter activity [29,30].

In the present study, we provide evidence that signaling through Gα12 to impact cancer cell invasion involves expression of cytokines IL-6, IL-8 as well as the metalloproteinase MMP-2. This activity of Gα12 leads to increased invasion not only of the cells expressing the activated G protein, but also of their neighboring cells due to the extracellular activities of the MMP and cytokines [31,32]. The end result of elevated Gα12 activity in the cells is an increase in the level of the cytokines and MMP in the media, and our evidence indicates that this is primarily a consequence of increased expression of these factors. Our findings indicate that transcriptional regulation is an important component, and that NF-κB activity is critical for the induction of promoter elements of these three secreted factors. This is not too surprising as earlier studies have linked G12 proteins and Rho activation to NF-κB activation [33], and Gα13-mediated VEGFR-2 expression has been reported to involve activation of RhoA and NF-κB [34]. However, we cannot exclude the involvement of additional transcription factors and/or secretory processes in cell invasion and metastasis promoted by activation of G12 proteins.

The involvement of Gα12 in tumor cell invasion hints at a connection between downstream signaling from this G protein and the process of epithelial-to-mesenchymal transition (EMT), which is characterized by loss of epithelial junctional markers and cell polarity and gain of mesenchymal proteins [35]. In this regard, several lines of investigation implicate Gα12 signaling in the regulation of cell shape change, cell adhesion and migration. In particular, expression of a constitutively active mammalian Gα12 in cultured cells was shown to trigger the formation of actin stress fibers, as well as assembly of focal adhesion complexes [18]. Activated Gα12 evoked cell rounding [36], and signaling through the sphingosine-1-phosphate (S1P) receptor was shown to stimulate stress fiber formation in a Gα12-dependent manner [37]. Expression of active Gα12 stimulated decreased phosphorylation of the focal adhesion proteins paxillin, focal adhesion kinase, and Crk-associated substrate [38,39]. Gα12 interacts with several members of the cadherin family, with eventual disruption of E-cadherin/β-catenin complex [11]. Furthermore, Gα12 was shown to negatively regulate epithelial cell attachment and migration on collagen-I by inhibiting α2β1 integrin function [39]. Finally, microarray studies reveal many EMT markers to be concomitantly up-regulated (e.g. vimentin, paxillin, IQGAP1) or down-regulated (E-cadherin, Radixin) in breast cancer cell lines expressing constitutively-active Gα12 [8]. Together, these data suggest a direct role of Gα12 in EMT. Since the process of EMT requires secretion of cytokines and metalloproteinases [32,40], our findings on induction of MMP-2 as well as IL-6 and IL-8 further support a potential involvement of Gα12 signaling in EMT.

The diversity of downstream targets of activated Gα12 has created a complex maze of potential signaling mechanisms and regulatory relationships between different effector proteins. Our observations identify novel factors of G12 signaling that may contribute to the regulation of breast cancer invasion and provide useful targets in therapeutic strategy. Although the evidence is compelling that Gα12 can induce oncogenic transformation of cells, as well as initiate progression of cancer cells toward metastasis, it is important to note that activating mutations in Gα12 in human tumors has not yet been reported. A broader analysis of human tumor samples, as well as more elaborate animal models of G12 deficiency and hyperactivity [41], will be essential for assessing the significance of Gα12 in the pathophysiology of human cancers and potentially other pathophysiologies.

## Conclusions

The capacity of activated Gα12 to impact cancer cell invasion involves its ability to increase expression of the cytokines IL-6 and IL-8 and the metalloproteinase MMP-2. The resultant increase in the level of the factors in media enhances invasion, not only of the cells exhibiting elevated levels of Gα12 activity, but also of neighboring cells.

## Methods

### Cell lines and transfections

MDA-MB-231 cells were routinely cultured in DMEM supplemented with 10% (v/v) fetal calf serum and 100 units/ml penicillin. MCF10A cells were routinely cultured in DMEM/F12 containing 20 ng/ml recombinant human epidermal growth factor (iDNA), 0.5 mg/ml hydrocortisone (Sigma-Aldrich, #H0396), 10 μg/ml insulin (Sigma-Aldrich, #I6634) and supplemented with 10% (v/v) fetal calf serum and 100 units/ml penicillin. Culture media, fetal bovine serum and penicillin were purchased from Invitrogen. To generate the MDA-MB-231 cell line stably expressing an inducible Gα12QL, the Tet-On 3G Inducible Expression System (Clontech) and the Lenti-X Lentiviral Expression System (Clontech) were employed following the manufacturer’s protocol. Doxycycline (1 μg/ml) was used to induce the expression of Gα12QL in the resulting MDA-MB-231 cell line.

MDA-MB-231 and MCF10A cells were plated at 200,000 cells per well in 6-well plates 24 h prior to transfection. Transfections were performed with pDNA3.1 (Mock), pcDNA3.1-mRFP (Addgene), dominant active Gα12 (Gα12QL) pcDNA3.1 [42], or an IRES construct expressing both GFP and Gα12QL cDNA in pLL-5.5 [43], using Attractene® (Qiagen). The cells were then transfected twice with 1 μg DNA in each transfection at a 24-hour interval. After another 24 h, the cells were washed twice with PBS then incubated at 37°C overnight in knockout (KO) DMEM (Invitrogen). The cells and media were then harvested and collected respectively for use in subsequent assays.

### Screening for cytokine and MMP secretion by protein array

Secretion of various cytokines and MMPs into media from cells expressing Gα12QL or mock vector was evaluated using three commercial arrays, Quantibody Human Inflammation Array-3, Quantibody Human TH1/TH2 Array-1 and Quantibody Human MMP Array-1 (RayBiotech, Inc. (Norcross, GA) according to the manufacturer’s protocol. These multiplexed technologies allowed simultaneously detection of 40 different cytokines.

For analysis of secreted proteins, media collected from cells were concentrated on an Amicon Ultra-4 Centrifugal Filter Unit (Millipore). Proteins were resolved by 10% SDS PAGE, electroblotted to PDVF membrane then probed with antibodies to MMP-2 (EMD Biosciences), IL-6 (Genscript, USA) or IL-8 (Genscript, USA). Membranes were subsequently incubated with HRP-labeled secondary antibodies and blots were developed using the ECL chemiluminescence detection kit (GE Healthcare). Loading controls were performed by silver staining of corresponding samples using Pierce Silver Staining kit. Fluorescent signals were detected by a laser scanner (Axon GenePix; Molecular Devices, Sunnyvale, CA, U.S.A.) set at 555 nm excitation and 565 nm emission. To determine the relative concentrations of cytokines in the media, the densities of individual spots were measured using ImageJ software.

### Cell invasion assay and cell treatment

Cell invasion was assessed using the matrigel invasion system. Transwell chambers (8 μm pore size, polycarbonate filters, 6.5 mm diameter; Costar) in24-well plates were coated with 100 μl of polymerized matrigel (BD Matrigel). Cells to be analyzed were incubated overnight in serum-free DMEM (Invitrogen), resuspended in 250 μl of serum-free medium and then added to the upper chambers. After incubation at 37°C for 24 h, the invaded cells on the lower side were obtained by trypsinization and counted or subjected to FACS analysis (for experiments with GFP or RFP constructs). To assess invasion in the presence of inhibitors, cells were placed into the upper chamber with media that contained 1 μM MMP inhibitor (Millipore, GM6001 #CC1100), 2 μM NF-κB inhibitor (Merck, #BAY 11–7082), 1 μM AP-1 inhibitor (a gift of Tay Su Ling, this institution) or 1 μg IL-6 and IL-8 antibodies (Genscript, USA).

### Gelatin zymography

Gelatinase activity was evaluated by zymography. Aliquots of media were resolved on a 10% SDS-polyacrylamide gel containing 0.1% gelatin under a non-reducing conditions. Following electrophoresis, gels were washed twice with 2.5% Triton X-100 for 30 min to remove SDS and renature the MMP species in the gels. Gels were then incubated in developing buffer (10 mM Tris pH 7.5, 0.1% Triton-X-100, 1 mM CaCl_2_) overnight to induce gelatin lysis by renatured MMPs. The active MMPs were detected as clear bands.

### *In situ* zymography

Gelatin-Oregon Green 488 conjugate (1 mg/ml) (Molecular probes, #G13186) was mixed 1:1 with 1% agarose melted in 50 mM Tris–HCl, pH 7.4, containing 10 mM calcium chloride and 0.05% Brij 35. The liquid mixture was spread onto pre-warmed glass slides and allowed to gel at room temperature; slides with substrate layers that appeared to be non-homogenous were discarded. 1 × 10^3^ cells were serum-starved for 24 h, suspended in DMEM, and applied on top of the substrate film. A coverslip was placed on top of the cells to prevent the cells from drying up. Slides were kept in a horizontal position, and incubated in humidified, light-protected chambers at 37°C overnight. Lysis of the substrate was assessed by examination under a fluorescent microscope (Olympus IX71S1F3).

### Small-interfering (si) RNAs

siRNAs targeting MMP-2 were synthesized (Qiagen) against human sequences. siRNA constructs with no known homology to mammalian genes were used as negative controls (All Stars Negative Control, Qiagen). Transfections were performed according to the manufacturer’s instructions with 50 nM of individual siRNAs using HiPerFect (Qiagen) for 18–24 h.

### Reporter constructs and luciferase assay

Reporter assays for promoter activity were undertaken using a Dual-Luciferase Reporter Assay System (Promega, Madison, WI, USA) as stipulated in the manufacturer’s manual. Briefly, 24 h before transfection, MDA-MB-231 or MCF10A cells were plated into 24-well tissue culture dishes at 4 × 10^4^ cells/well. Transfections were performed in triplicate using Lipofectamine PLUS reagent (Invitrogen); 0.4 μg of reporter plasmids for MMP2-Luc, IL-6-Luc or IL-8-Luc (all Firefly luciferase) promoters were co-transfected with 10 ng of pRL (Renilla luciferase). Luciferase assays were performed 36 h after transfection. Firefly luciferase activity was normalized to Renilla luciferase readings in each well.

### Chromatin Immunoprecipitation (ChIP) Assay

Chip assays were performed following a standard protocol. Briefly, two million cells per sample were harvested, neutralized in 0.125 M glycine solution for 10 min, and subject to cross-linking with 4% formaldehyde for 10 min at room temperature. Sonication conditions (30 sec burst with 30 sec rest ×5 times on ice, 70% power at Diagenode BioRuptor™ Sonicator) were identified to ensure that all major DNA fragments were in the range of 250–400 bp. Disrupted samples were then incubated overnight with either NF-κB mAb (Cell Signaling) or non-immune control antibodies (anti-clathrin, AbCam). Samples were then precipitated using protein A-Agarose/Salmon Sperm DNA beads (Millipore, MA, USA), pellets were de-crosslinked with 1 M NaCl, 50°C) and DNA was purified using a PCR Isolation kit (Qiagen). The following pairs of primers were used for amplification of DNA from IP-samples: IL-8-NF-κBsite1: Forward ACATGCAGGCACTAATCTGGAAGC, Reverse GCATCTTTCTCCTCACTTTGGCCT; IL-8-NF-κBsite2: Forward AGTGTGATGACTCAGGTTTGCCCT, Reverse AGATGGTTCCTTCCGGTGGTTTCT; IL-8-NF-κB Off Target :Forward CAGCTGCTTGCTTGCTATGTGTGT, Reverse TGGCTATGGCTGGTGGTGGAAATA; IL-6-NF-κB: Forward TGCAGGAAATCCTTAGCCCTGGAA, Reverse TGCCGTCGAGGATGTACCGAATTT; IL-6-NF-κB Off-Target: Forward TCACCAGACCAAGGAGCTACAACA, Reverse GGAACTTGCAGTTGCTGCCAGAAT; MMP2 NF-κB_binding site: Forward ACGGTTGTCACAGGGAGAACTTCA, Reverse TTGGAACCAGAGCGACTCCATCTT; MMP2 NF-κB_binding sites Off Target: Forward AAGGGCCTAGAGCGACAGATGTTT, Reverse: GGTCCTGGCAATCCCTTTGTATGT.PCR amplification of genomic regions containing the putative NF-κB binding sites were performed in triplicate and the relative occupancy of the immunoprecipitated factor at a locus was estimated by using the comparative threshold method [44].

## Competing interests

The authors declare that they have no competing interests.

## Authors’ contributions

CYC conducted the majority of the experiments. UK carried out experiments on the impact of IL-6 and IL-8 blockade on G12-stimulated invasion, and was involved in DNA construct preparation and evaluation. PJC and Alex Lyakhovich conceived of the study, and participated in its design and coordination. CYC and PJC wrote the manuscript, and all authors read and approved the final manuscript.

## Supplementary Material

Additional file 1: Figure S1 Human TH1/TH2 array 1. **Figure S2.** Human MMP array 1. **Figure S3.** Human inflammation array 3. **Figure S4.** Expression of dominant active Dα12 in MCF10A cells induces secretion of cytokines IL-6 and IL-8, and MMP-2. **Figure S5.** Validation of increased secretion of IL-8 and MMP-2 expression of MCF10A cells expression dominant activate Gα12. **Figure S6.** Analysis of MMP-2 activity upon Gα12QL expression by *in-situ* zymography. **Figure S7.** Interleukins and MMP-2 are invoved in Gα12-mediated invasion of MCF10A cells. **Figure 8.** Schematic representation of the transcription factor binding sited presents in the the 5′ UTRs of the IL-6, IL-8, MMP-2 and MMP-9 promoters. Click here for file
