# Supplementary material for: Breast cancer cell invasion mediated by Gα12 signaling involves expression of interleukins-6 and −8, and matrix metalloproteinase-2
Source: J Mol Signal. 2014 Jun 17;9:6. doi: 10.1186/1750-2187-9-6 (PMC4074425; doi:10.1186/1750-2187-9-6)
Supplement: Additional file 1: Figure S1 — Human TH1/TH2 array 1. Figure S2. Human MMP array 1. Figure S3. Human inflammation array 3. Figure S4. Expression of dominant active Dα12 in MCF10A cells induces secretion of cytokines IL-6 and IL-8, and MMP-2. Figure S5. Validation of increased secretion of IL-8 and MMP-2 expression of MCF10A cells expression dominant activate Gα12. Figure S6. Analysis of MMP-2 activity upon Gα12QL expression by in-situ zymography. Figure S7. Interleukins and MMP-2 are invoved in Gα12-mediated invasion of MCF10A cells. Figure 8. Schematic representation of the transcription factor binding sited presents in the the 5′ UTRs of the IL-6, IL-8, MMP-2 and MMP-9 promoters. [file 1750-2187-9-6-S1.pptx]

## Slide 1
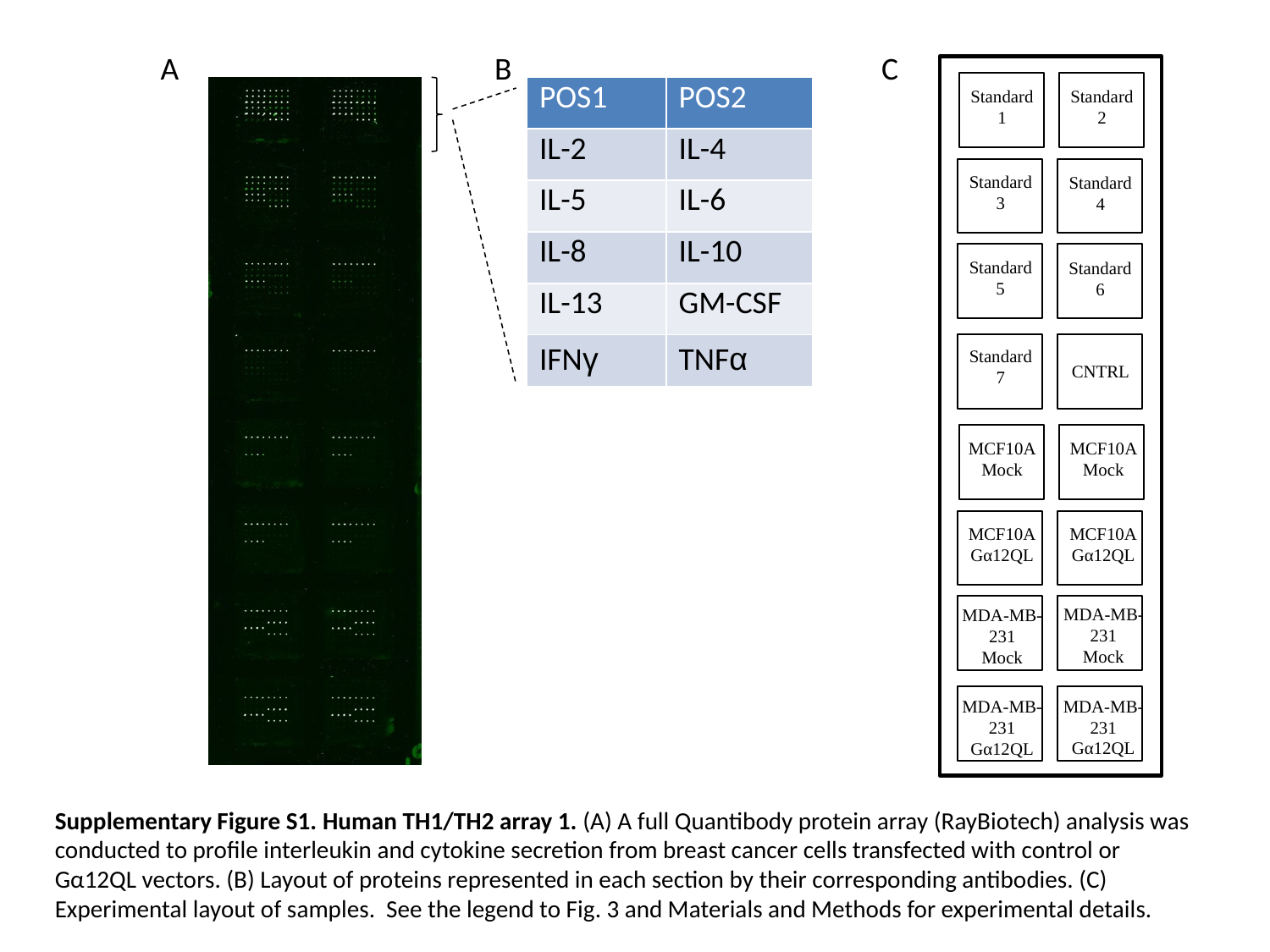

A B			 C
Standard
1
Standard
2
Standard
3
Standard
4
Standard
5
Standard
6
Standard
7
CNTRL
MCF10A
Mock
MCF10A
Mock
MCF10A
Gα12QL
MCF10A
Gα12QL
MDA-MB-231
Mock
MDA-MB-231
Mock
MDA-MB-231
Gα12QL
MDA-MB-231
Gα12QL
| POS1 | POS2 |
| --- | --- |
| IL-2 | IL-4 |
| IL-5 | IL-6 |
| IL-8 | IL-10 |
| IL-13 | GM-CSF |
| IFNγ | TNFα |
Supplementary Figure S1. Human TH1/TH2 array 1. (A) A full Quantibody protein array (RayBiotech) analysis was conducted to profile interleukin and cytokine secretion from breast cancer cells transfected with control or Gα12QL vectors. (B) Layout of proteins represented in each section by their corresponding antibodies. (C) Experimental layout of samples. See the legend to Fig. 3 and Materials and Methods for experimental details.

## Slide 2
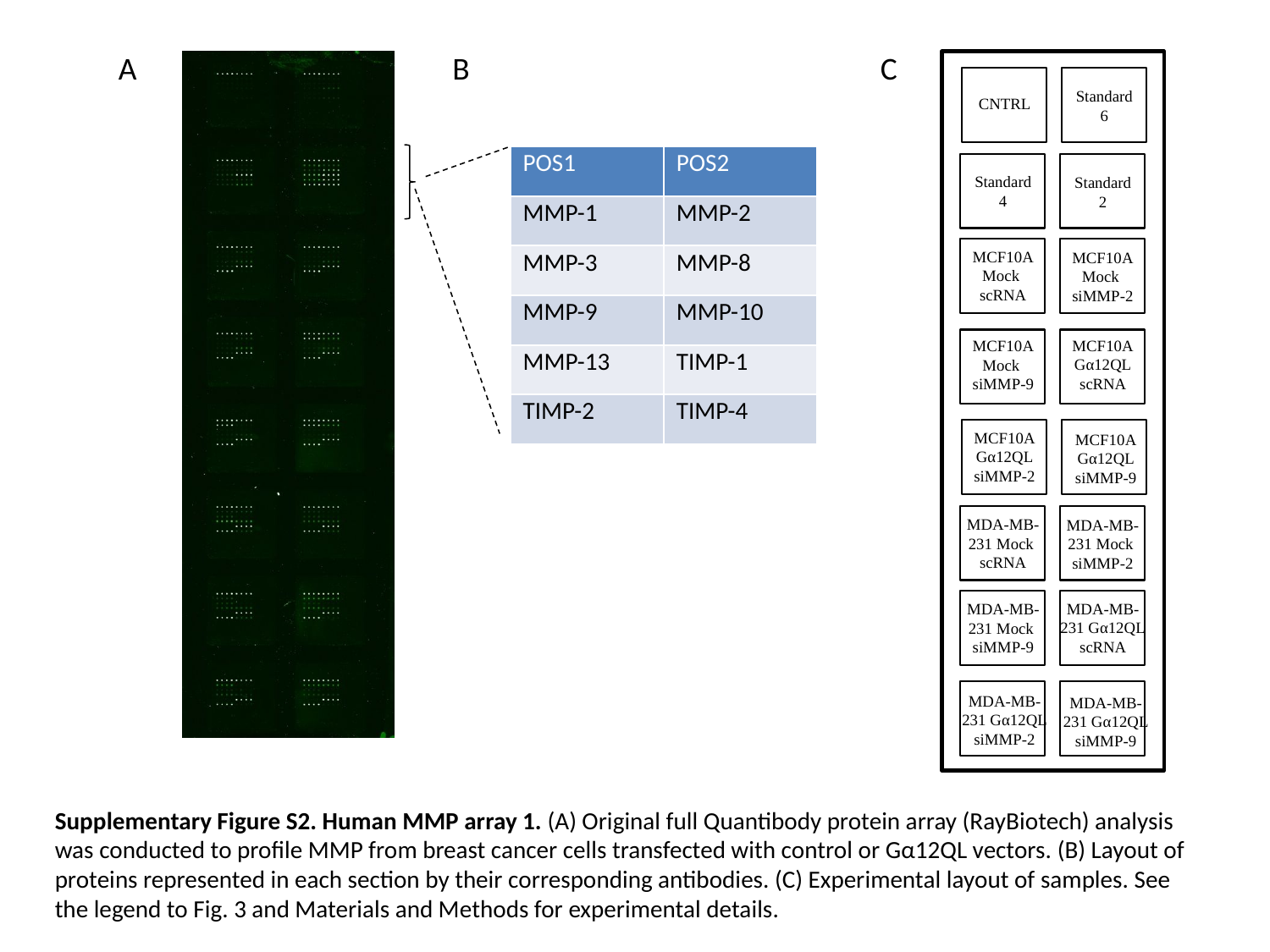

A B				C
Standard
6
CNTRL
Standard
4
Standard
2
MCF10A
Mock
scRNA
MCF10A
Mock
siMMP-2
MCF10A
Gα12QL
scRNA
MCF10A
Mock
siMMP-9
MCF10A
Gα12QL
siMMP-2
MCF10A
Gα12QL
siMMP-9
MDA-MB-231 Mock
scRNA
MDA-MB-231 Mock
siMMP-2
MDA-MB-231 Gα12QL
scRNA
MDA-MB-231 Mock
siMMP-9
MDA-MB-231 Gα12QL
siMMP-2
MDA-MB-231 Gα12QL
siMMP-9
| POS1 | POS2 |
| --- | --- |
| MMP-1 | MMP-2 |
| MMP-3 | MMP-8 |
| MMP-9 | MMP-10 |
| MMP-13 | TIMP-1 |
| TIMP-2 | TIMP-4 |
Supplementary Figure S2. Human MMP array 1. (A) Original full Quantibody protein array (RayBiotech) analysis was conducted to profile MMP from breast cancer cells transfected with control or Gα12QL vectors. (B) Layout of proteins represented in each section by their corresponding antibodies. (C) Experimental layout of samples. See the legend to Fig. 3 and Materials and Methods for experimental details.

## Slide 3
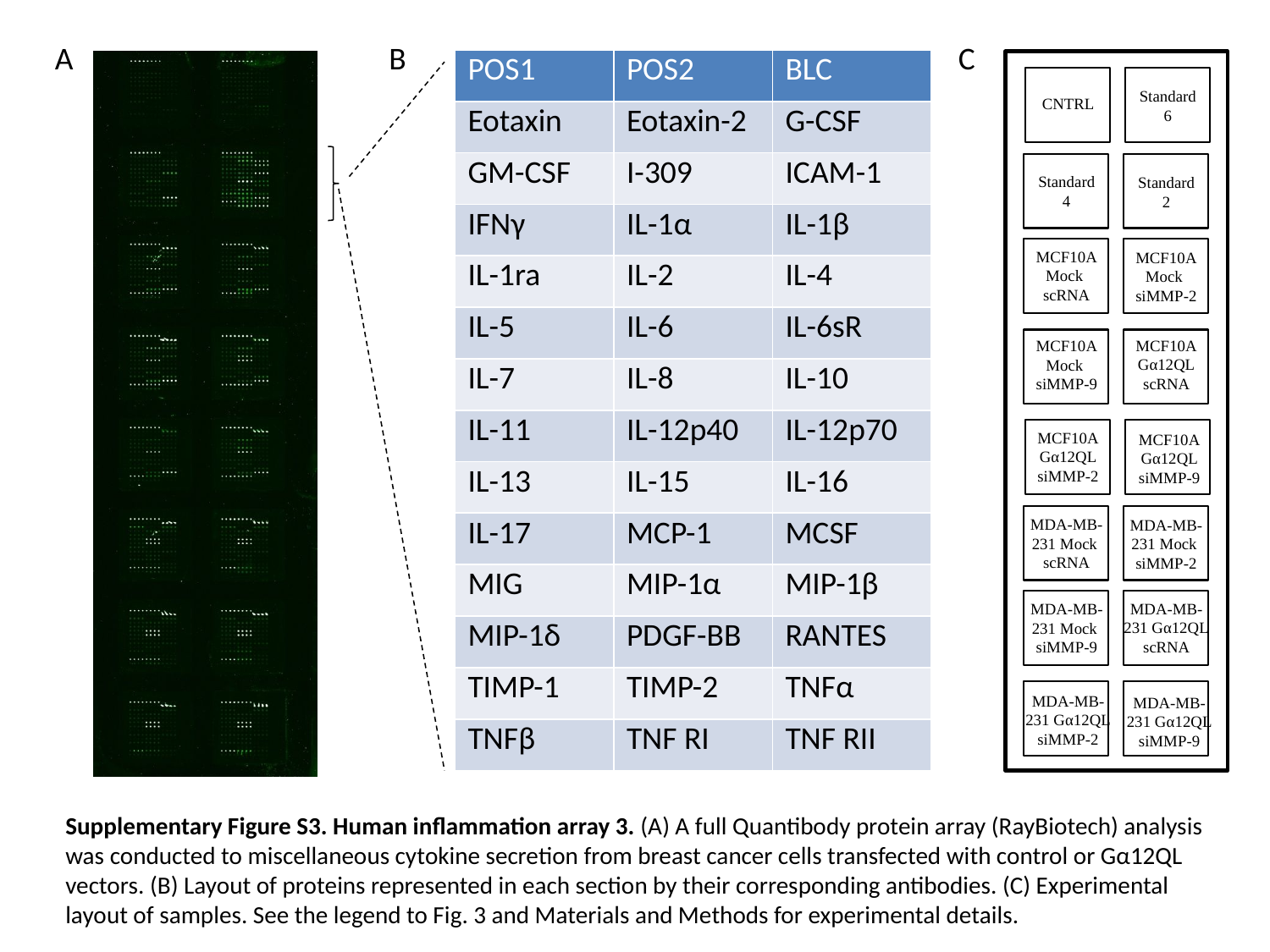

A B					 C
| POS1 | POS2 | BLC |
| --- | --- | --- |
| Eotaxin | Eotaxin-2 | G-CSF |
| GM-CSF | I-309 | ICAM-1 |
| IFNγ | IL-1α | IL-1β |
| IL-1ra | IL-2 | IL-4 |
| IL-5 | IL-6 | IL-6sR |
| IL-7 | IL-8 | IL-10 |
| IL-11 | IL-12p40 | IL-12p70 |
| IL-13 | IL-15 | IL-16 |
| IL-17 | MCP-1 | MCSF |
| MIG | MIP-1α | MIP-1β |
| MIP-1δ | PDGF-BB | RANTES |
| TIMP-1 | TIMP-2 | TNFα |
| TNFβ | TNF RI | TNF RII |
Standard
6
CNTRL
Standard
4
Standard
2
MCF10A
Mock
scRNA
MCF10A
Mock
siMMP-2
MCF10A
Gα12QL
scRNA
MCF10A
Mock
siMMP-9
MCF10A
Gα12QL
siMMP-2
MCF10A
Gα12QL
siMMP-9
MDA-MB-231 Mock
scRNA
MDA-MB-231 Mock
siMMP-2
MDA-MB-231 Gα12QL
scRNA
MDA-MB-231 Mock
siMMP-9
MDA-MB-231 Gα12QL
siMMP-2
MDA-MB-231 Gα12QL
siMMP-9
Supplementary Figure S3. Human inflammation array 3. (A) A full Quantibody protein array (RayBiotech) analysis was conducted to miscellaneous cytokine secretion from breast cancer cells transfected with control or Gα12QL vectors. (B) Layout of proteins represented in each section by their corresponding antibodies. (C) Experimental layout of samples. See the legend to Fig. 3 and Materials and Methods for experimental details.

## Slide 4
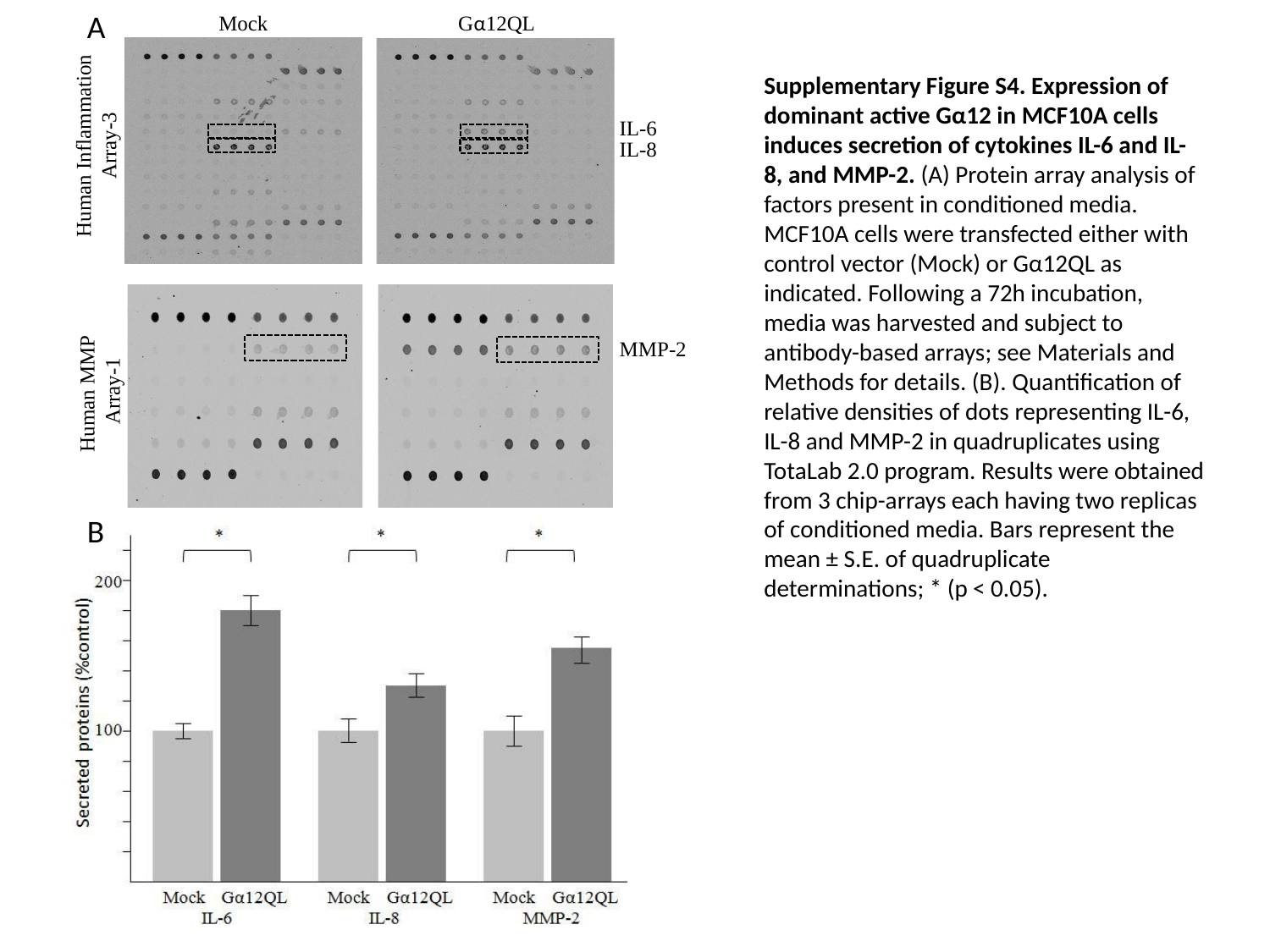

A
B
 Mock Gα12QL
Supplementary Figure S4. Expression of dominant active Gα12 in MCF10A cells induces secretion of cytokines IL-6 and IL-8, and MMP-2. (A) Protein array analysis of factors present in conditioned media. MCF10A cells were transfected either with control vector (Mock) or Gα12QL as indicated. Following a 72h incubation, media was harvested and subject to antibody-based arrays; see Materials and Methods for details. (B). Quantification of relative densities of dots representing IL-6, IL-8 and MMP-2 in quadruplicates using TotaLab 2.0 program. Results were obtained from 3 chip-arrays each having two replicas of conditioned media. Bars represent the mean ± S.E. of quadruplicate determinations; * (p < 0.05).
IL-6
IL-8
 Human Inflammation
Array-3
MMP-2
Human MMP
Array-1

## Slide 5
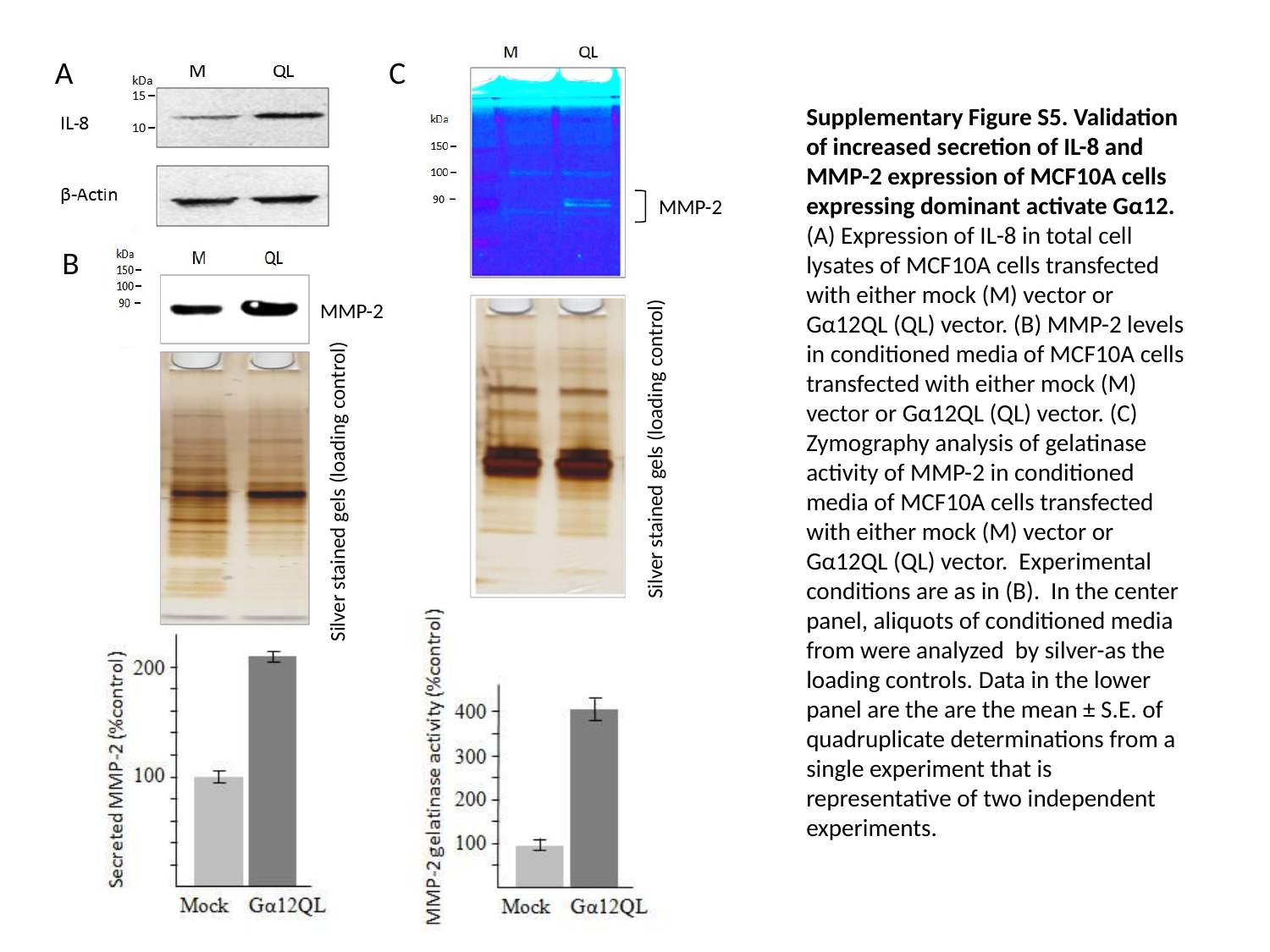

A C
 B
Supplementary Figure S5. Validation of increased secretion of IL-8 and MMP-2 expression of MCF10A cells expressing dominant activate Gα12. (A) Expression of IL-8 in total cell lysates of MCF10A cells transfected with either mock (M) vector or Gα12QL (QL) vector. (B) MMP-2 levels in conditioned media of MCF10A cells transfected with either mock (M) vector or Gα12QL (QL) vector. (C) Zymography analysis of gelatinase activity of MMP-2 in conditioned media of MCF10A cells transfected with either mock (M) vector or Gα12QL (QL) vector. Experimental conditions are as in (B). In the center panel, aliquots of conditioned media from were analyzed by silver-as the loading controls. Data in the lower panel are the are the mean ± S.E. of quadruplicate determinations from a single experiment that is representative of two independent experiments.
MMP-2
MMP-2
Silver stained gels (loading control)
Silver stained gels (loading control)

## Slide 6
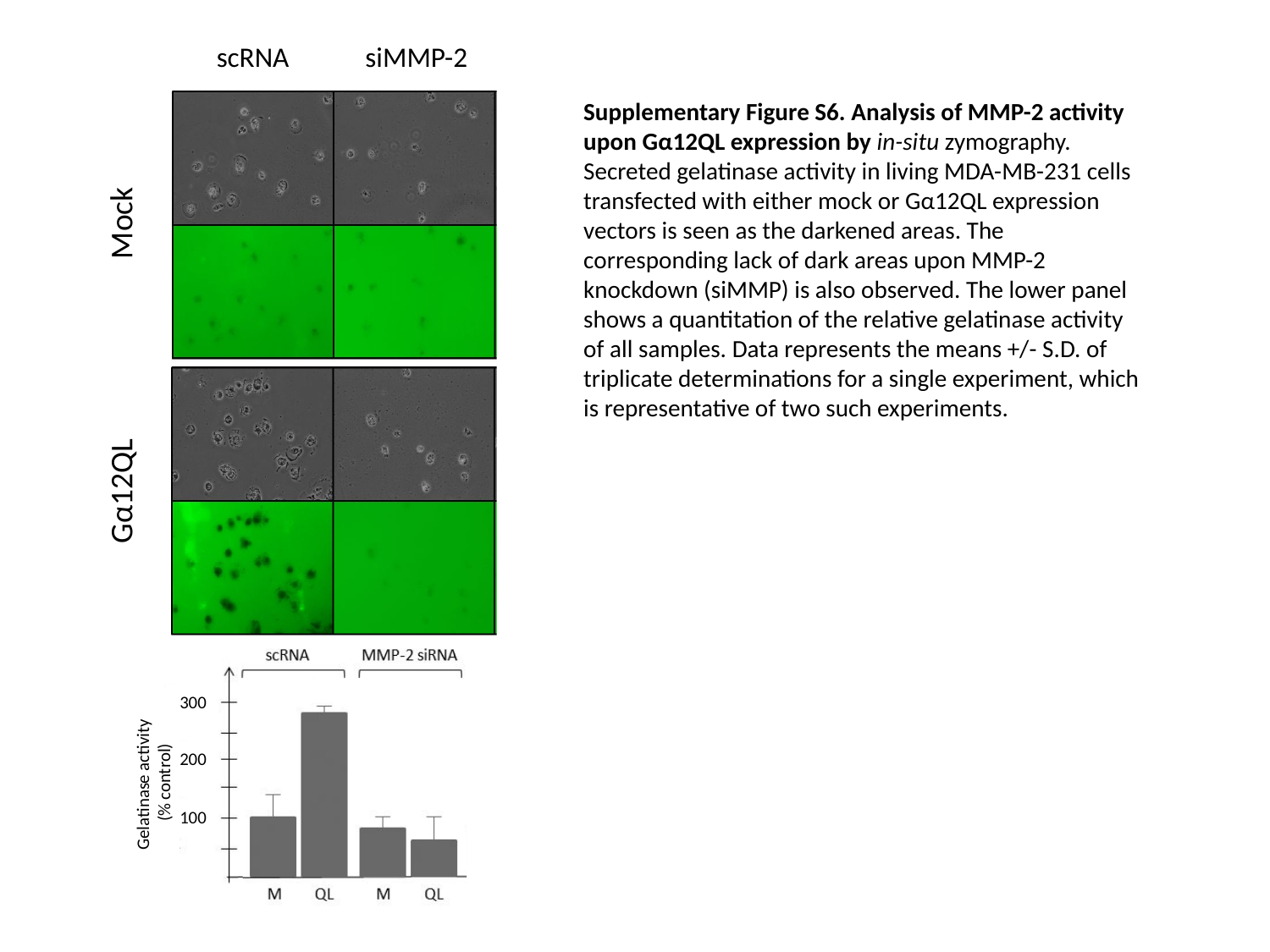

scRNA siMMP-2
 Gα12QL Mock
300
200
Gelatinase activity
(% control)
100
Supplementary Figure S6. Analysis of MMP-2 activity upon Gα12QL expression by in-situ zymography. Secreted gelatinase activity in living MDA-MB-231 cells transfected with either mock or Gα12QL expression vectors is seen as the darkened areas. The corresponding lack of dark areas upon MMP-2 knockdown (siMMP) is also observed. The lower panel shows a quantitation of the relative gelatinase activity of all samples. Data represents the means +/- S.D. of triplicate determinations for a single experiment, which is representative of two such experiments.

## Slide 7
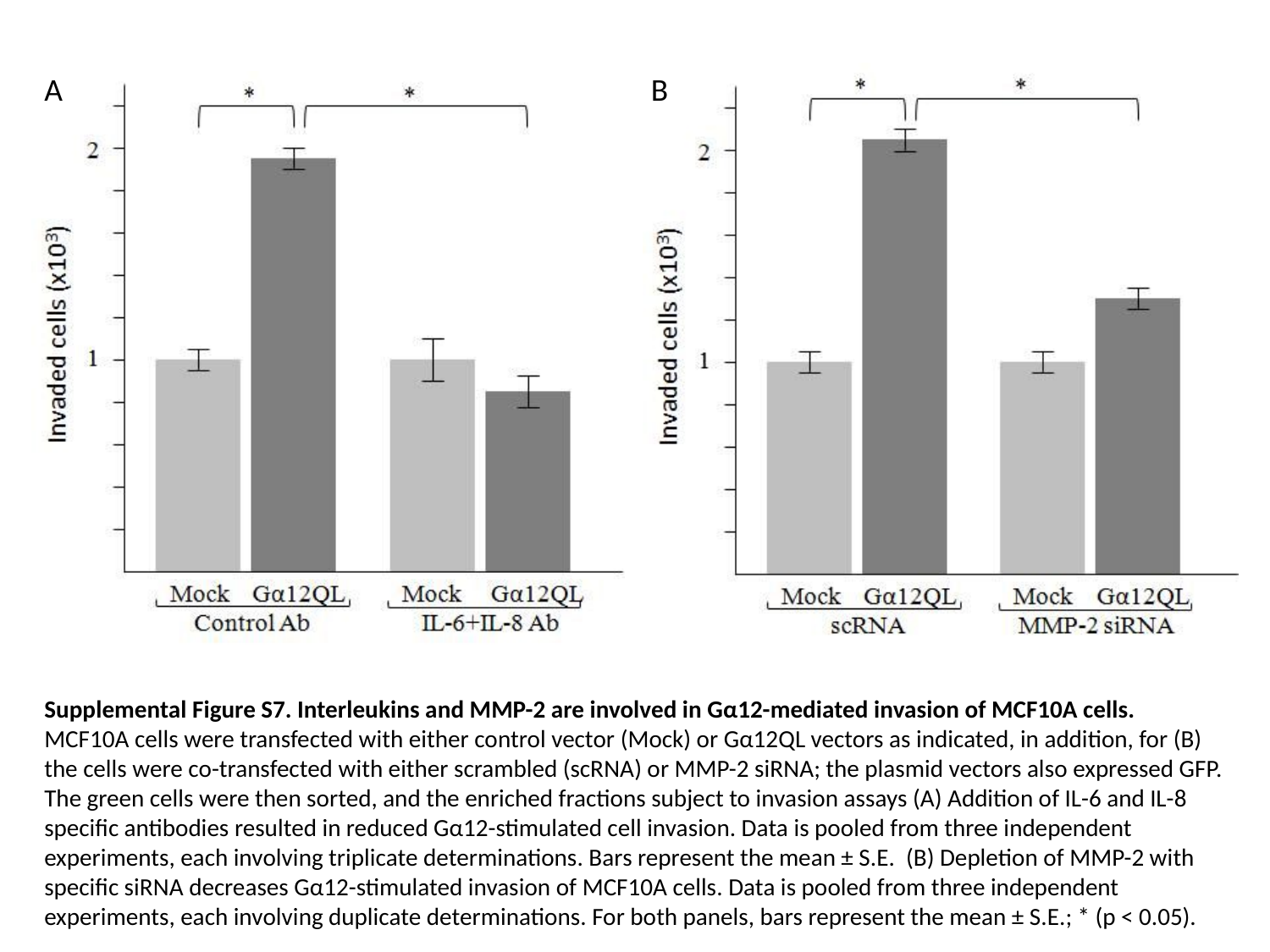

A B
Supplemental Figure S7. Interleukins and MMP-2 are involved in Gα12-mediated invasion of MCF10A cells.
MCF10A cells were transfected with either control vector (Mock) or Gα12QL vectors as indicated, in addition, for (B) the cells were co-transfected with either scrambled (scRNA) or MMP-2 siRNA; the plasmid vectors also expressed GFP. The green cells were then sorted, and the enriched fractions subject to invasion assays (A) Addition of IL-6 and IL-8 specific antibodies resulted in reduced Gα12-stimulated cell invasion. Data is pooled from three independent experiments, each involving triplicate determinations. Bars represent the mean ± S.E. (B) Depletion of MMP-2 with specific siRNA decreases Gα12-stimulated invasion of MCF10A cells. Data is pooled from three independent experiments, each involving duplicate determinations. For both panels, bars represent the mean ± S.E.; * (p < 0.05).

## Slide 8
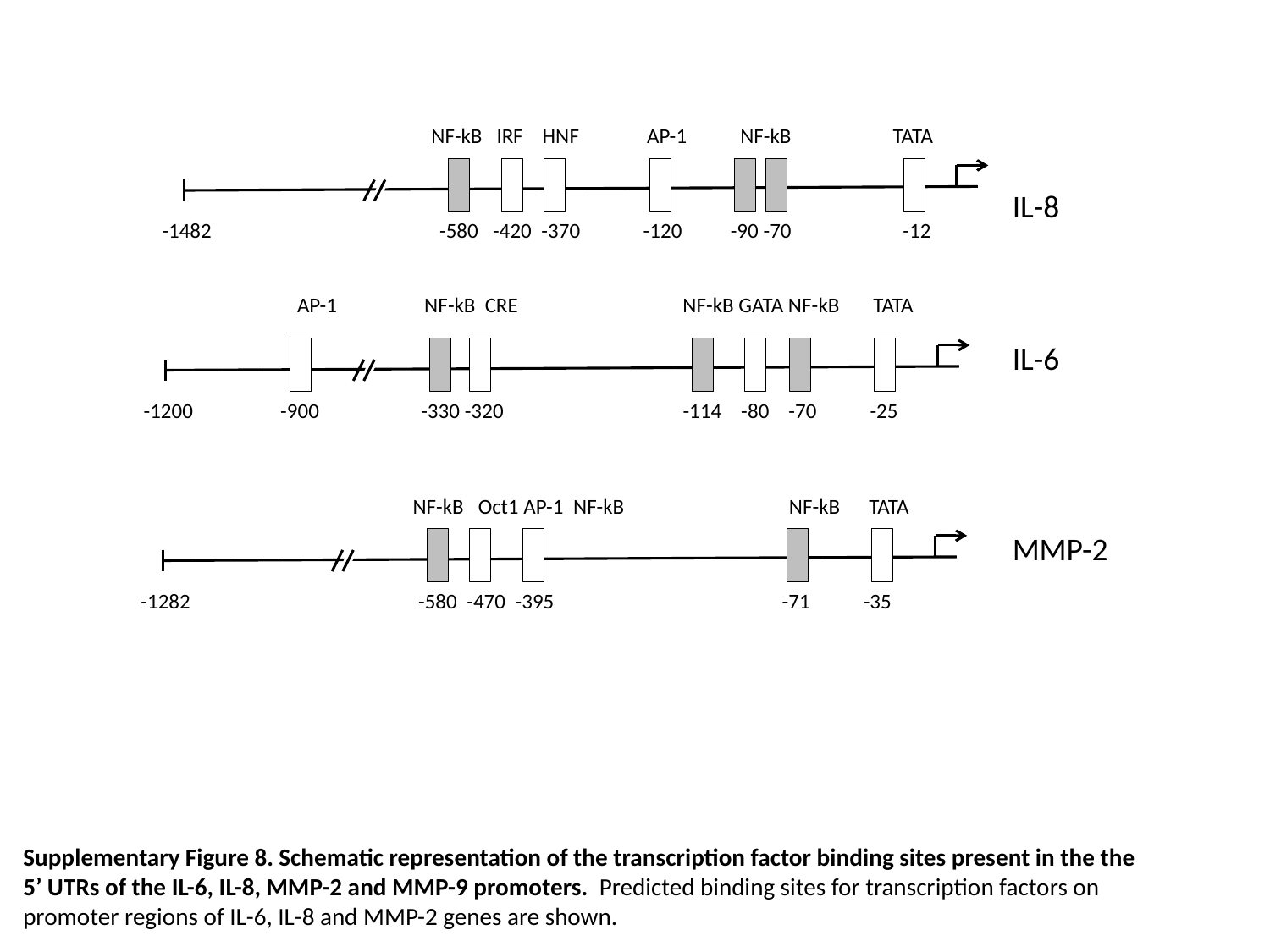

NF-kB IRF HNF AP-1 NF-kB TATA
IL-8
IL-6
MMP-2
-1482 -580 -420 -370 -120 -90 -70 -12
AP-1 NF-kB CRE NF-kB GATA NF-kB TATA
-1200 -900 -330 -320 -114 -80 -70 -25
NF-kB Oct1 AP-1 NF-kB NF-kB TATA
-1282 -580 -470 -395 -71 -35
Supplementary Figure 8. Schematic representation of the transcription factor binding sites present in the the 5’ UTRs of the IL-6, IL-8, MMP-2 and MMP-9 promoters. Predicted binding sites for transcription factors on promoter regions of IL-6, IL-8 and MMP-2 genes are shown.
